# The Presence of Selected Elements in the Microscopic Image of Pine Needles as an Effect of Cement and Lime Pressure within the Region of Białe Zagłębie (Central Europe)

**DOI:** 10.3390/toxics9010015

**Published:** 2021-01-19

**Authors:** Mirosław Szwed, Witold Żukowski, Rafał Kozłowski

**Affiliations:** 1 Institute of Geography and Environmental Sciences, Jan Kochanowski University, 25-406 Kielce, Poland; rafal.kozlowski@ujk.edu.pl; 2 Faculty of Chemical Engineering and Technology, Cracow University of Technology, 31-155 Cracow, Poland; witold.zukowski@pk.edu.pl

**Keywords:** air pollution, biomonitoring, trace elements, SEM/EDS

## Abstract

In this study, we present the results of microscopic observations of pine needles *Pinus sylvestris* L. collected in the area of cement-lime pressure in the south-western part of the Świętokrzyskie Mountains in the region of Białe Zagłębie. Images of scanning electron microscopy (SEM) confirm the presence of particles with a size of about 2 to 20 µm on the surface of the needles. Analysis using X-ray energy dispersion spectroscopy (EDS) allowed, in turn, to identify lead, iron, aluminium, calcium, and silicon in particles deposited in the surface layer of assimilation organs and dispersed in the surface layer of vegetation tissue within cell structures. Chemical composition, size and shape of particles of foreign bodies on the needles’ surface allow them to be identified as cement-lime dust coming from production plants located in the Białe Zagłębie. Negative influence on the condition and liveliness of Scots pine in the study area is manifested by images on which stomata is sealed, which limits the possibility of gas exchange.

## 1. Introduction

Decades of years of cement and lime plant operations in the south-western part of the Świętokrzyskie Mountains cause multi-directional anthropogenic pressure on the environment. Periodically repeatable exceedances of permissible concentration limitations of some pollutants, especially cement and lime dust [1], may cause a threat to human life and the existence of the natural environment [2,3,4]. In the 1970s, the Białe Zagłębie [5] covered an area of over 960 km^2^. Currently, raw material exploitation on an industrial scale concentrating in its area [6] amounts to approximately 180 km^2^. The size and structure of activity in this region has been significantly transformed over the half-century of its operation. Out of several local quarries and small factories operating at the end of the 20th century, only a few have remained, cooperating with existing cement plants in Nowiny and Małogoszcz as well as limestone plants in Bukowa and Trzuskawica [7]. 

Research on the environmental impact of the plants located in the Białe Zagłębie has been conducted by many researchers, so far. They have indicated the impact of alkaline dust on soils and vegetation [8,9], rain water [10,11,12], as well as changes in the aquatic environment caused by mining activities [13]. Research done dealing with the circulation of heavy metals in the environment draws attention to the potential threat arising from their toxicity and harmfulness to living organisms and their surroundings [14,15]. 

Research on the effects of cement dust on vegetation has shown a negative impact on vegetation growth and development by blocking incident solar radiation, leaves’ overheating, obstruction of stomata, and reducing the rate of photosynthesis [16,17,18]. The effect of capturing solid particles by stomata or epidermal fissures is the accumulation in the tissues of assimilation organs of both essential and insignificant minerals [19,20,21]. Material in the form of alkaline particles may also affect the course of biological processes [22,23]. The presence of solid particles in the atmosphere is the result of the entire cement production cycle, i.e., from the extraction of limestone to its packaging and shipment [24,25]. The basic component of cement dust is calcium [10,26], but it may also contain heavy metals such as zinc, copper, iron, and even more toxic ones, namely cadmium, lead, and chromium [10,27,28,29,30].

The IARC (International Agency of Research on Cancer) confirmed, in turn, the carcinogenic effects of cadmium, nickel, and chromium compounds [31]. The presence of these heavy metals in cement-lime dust poses a potential threat to human life and the environment. This research focuses on the impact of the cement and lime industry on forest resources. The area of Białe Zagłębia, with an area of about 200 km^2^, has a forest coverage of 32% [32]. Scots pine (*Pinus sylvestris*) is the forest-forming species. Industrialized areas, including mines, occupy about 3%. Despite the large difference in both areas, the influence of the majority of industries on forest resources is significant.

The paper identifies sources of dust pollution with the use of SEM/EDS techniques and assesses their impact on the condition of forest resources within the Białe Zagłebie.

Pine needles in the study area are a very good bio-indicator of air pollution, especially dust. This results from their properties [33]. Due to high concentrations of heavy metals in cement and lime dust, it may be assumed that the chemical composition of needles is essentially affected by emitted cement and lime dust. Of course, soil chemistry also has an impact on their composition. Nevertheless, due to soil alkalinity in this area, the role of this source is not dominant, and this results from the low solubility of heavy metals in these conditions. As Parzych et al. [34] note, it is an increase in soil acidity that affects the bioavailability of selected heavy metals (Mn, Fe) in pine needles.

## 2. Materials and Methods 

Due to the prevalence of Scots pine *Pinus sylvestris* L. in the study area, forming dense complexes of upland and lowland forests with rich undergrowths and lower fir floors, their needles were selected for microscopic analyses [32]. Two-year needles are considered to be the best for research, because the elder ones, especially those existing in a contaminated environment, may already be damaged and their chemical composition may change [35,36].

**Figure 1:**
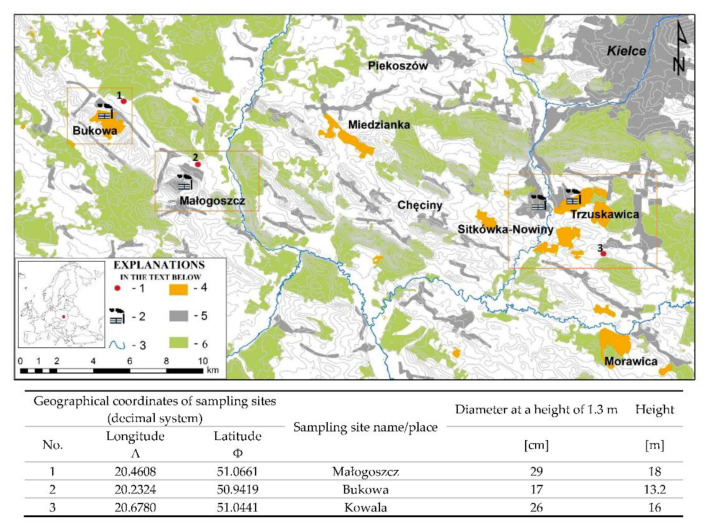
Location of study area (elaborated by M. Szwed), 1—sampling points, 2—cement and limestone plants, 3—rivers, 4—quarries, 5—buildings, 6—forests.

Scots pine *Pinus sylvestris* L. needles’ samples were collected in 2018 in Bukowa (limestone plant), Małogoszcz (cement plant), and Kowala (vicinity of the cement plant in Nowiny and the limestone plant in Trzuskawica) in an amount of approximately 200 g for each sampling site. The research sample consisted of mixed and averaged samples collected from about eight trees of the same age at each designated sampling site (Figure 1). The diameter of each tree was about 25 cm. The needles were taken from the whorl, on the top of tree, from the most exposed places possible.

Samples collected in the field were analysed at the Environmental Research Laboratory of the Jan Kochanowski University in Kielce. After washing the samples three times with deionized water, they were dried at 65 °C in order to obtain an air-dry mass. In the ground samples, pH was determined in a solution of water and 1N KCl in a volume ratio of 1:2.5. In order to determine the chemical composition of heavy metals, the samples were mineralised in the Multiwave 3000 Aanton Paar mineraliser. For this purpose, a sample containing 0.1 g was weighed and mineralised with nitric acid V (Suprapur Merck 65%) and perhydrol (30%) in a volume ratio of 2.5:1 (microwave power: 1400 W, temperature: 2000 °C, time: 40 min). After mineralisation, the samples were analysed using the ICP-MS-TOF OptiMass 9500 mass spectrometer (GBC Scientific Equipment, Melbourne, Australia). In order to control the quality of obtained results, certified reference materials such as ERM-CA713 were used.

Scanning electron microscopy (SEM) images along with the X-ray energy dispersive spectroscopy (EDS) analysis were made using a TM3000 microscope (Hitachi, Tokyo, Japan) with a 15 kV accelerating voltage at the Institute of Chemistry and Inorganic Technology of the Cracow University of Technology.

## 3. Results

### 3.1. Chemical Analysis

As a result of the operations of the cement and lime plants in the Białe Zagłębie, 292.8 tonnes of cement-lime dust, 1402.2 tonnes of sulphur dioxide and 2548 tonnes of nitrogen oxides were emitted into the atmosphere in 2018 [unpublished data of Provincial Inspectorate of Environmental Protection in Kielce]. In the same year, the measuring stations of the Provincial Inspectorate for Environmental Protection in Nowiny and Małogoszcz registered 89 and 40 days, respectively, exceeding permissible standards for suspended particulates [37].

Chemical analysis of dust samples collected in such cement plants as Dyckerhoff Nowiny and Lafarge Małogoszcz carried out in the Environmental Research Laboratory in Kielce, using the ICP-MS/TOF OptiMass 9500 atomic adsorption spectrometer, indicates a significant share of heavy metals (Table 1). The largest share was reported for iron, aluminium, and magnesium, constituting over 97% of all analysed elements in both samples. Then, there were zinc, strontium, manganese, and lead, whose content was amounting to about 2%. The pH of both samples was alkaline, its value in H_2_0 and KCl was pH_H20_ = 13.21 and pH_KCl_ = 13.02 for the sample from Małogoszcz and pH_H20_ = 12.61 and pH_KCl_ = 12.38 for the sample from Nowiny.

As far as the dust sample from the cement plant in Małogoszcz is concerned, the descending sequence of marked heavy metals was as follows: Al > Fe > Mg > Zn > Sr > Pb > Mn > Ni > Cr > Cu > Co > Cd. When it comes to the dust sample collected from the cement plant in Nowiny, the order of the first four items remains unchanged Al > Fe > Mg > Zn, and afterwards—Mn > Sr > Pb > Ni > Cr > Cu > Co > Cd. 

The highest values of accumulated heavy metals in pine needles were recorded in the case of iron (245.9 mg·kg^−1^ d.m.), manganese (196.7 mg·kg^−1^ d.m.), aluminium (98.7 mg·kg^−1^ d.m.), zinc (55.3 mg·kg^−1^ d.m.), and copper (15.75 mg·kg^−1^ d.m.).

### 3.2. SEM/EDS Analysis

**Figure 2:**
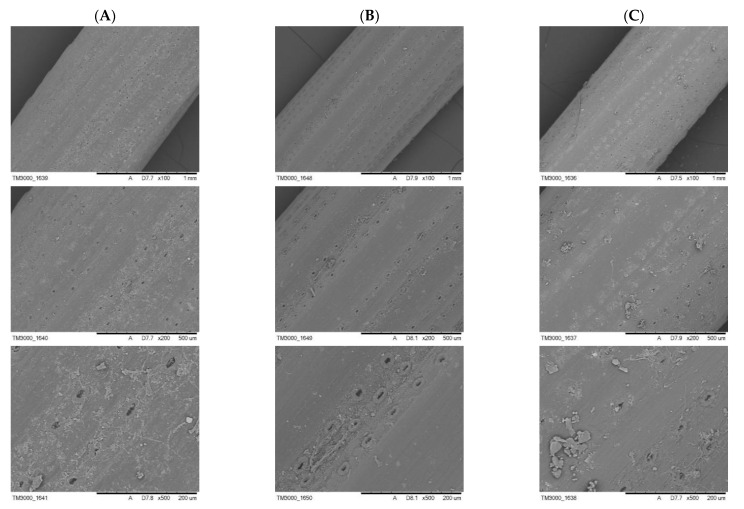
Scanning electron microscopy (SEM) microscopic images of pine needles; (**A**) Małogoszcz, (**B**) Bukowa, (**C**) Kowala.

**Figure 3:**
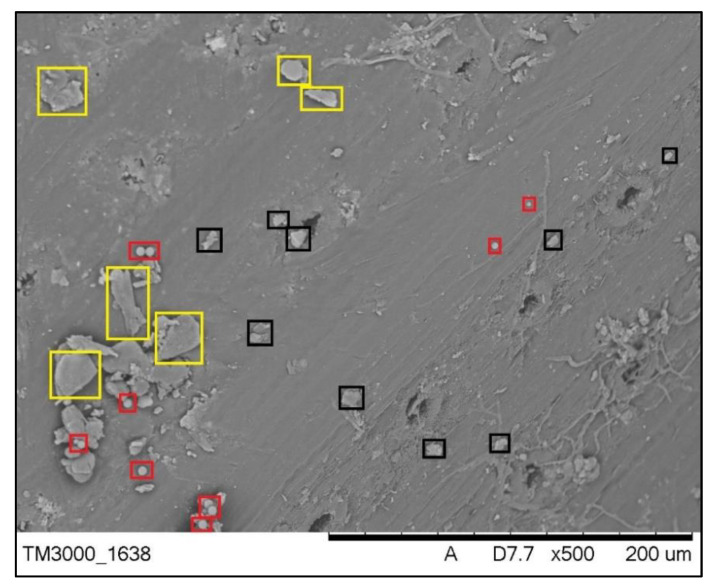
SEM microscopic images with marked amorphous and spherical particles on the surface of a *Pinus sylvestris* L. pine’s needle from Kowala.

Microscopic images of the surface of needles collected at a distance of 0.5 km east of the cement plant in Małogoszcz confirmed the presence of a variety of solid particles (Figure 2). Some of them had a spherical shape and sizes from 1 to 5 µm. In addition, irregularly shaped particles with a size of approximately 10 to 20 µm were present within the stomata (Figure 3). In the case of finer particles, their size allows free penetration of needles through the stomata. Stomata holes have different sizes, their lengths reach 20 to 30 µm, and their widths may be estimated at 5 to 10 µm. Therefore, particles that are <5 µm freely fall into the stomata. In a single stomata apparatus, the presence of one to several spherical particles of different sizes may be observed. Their presence causes the sealing of stomata and thus makes gas exchange more difficult. Similar observations may be made for the images obtained for the needles collected in the vicinity of the limestone plants in Bukowa and Kowala. In the images of needles collected in Bukowa, finer dust dominated; while in the case of Kowala, the presence of larger particles of irregular shape was more significant. 

The images also indicated a variable number of rows of stomata, i.e., 11 in Małogoszcz, 12 in Bukowa, and 10 in Kowala. The distance between rows was also variable (the smallest one in Bukowa, i.e., about 40 µm, and the largest one in Kowala—about 80 µm).

Three groups of particles were identified on the surface of needles, characterised by different morphological characteristics (Figure 3). The first group of components consist of irregularly shaped particles with a variable size of approximately 10 to 20 µm (marked with black colour). They are commonly found in dust floating freely in the air, arising naturally as a result of weathering rocks and minerals. Gypsum particles are much larger. They result from the burning of limestone in kilns fired with fuel of high content of sulphur (marked with a yellow colour). At the same time, dust containing significant amounts of calcium under the influence of water, in the presence of sulphur contained in the air or in the dust itself, causes the formation of hydrated calcium sulphate—gypsum. The third group consists of particles of much smaller dimensions (<5 µm), characterised by a spherical shape that they owe to high temperature transformations in the presence of iron [38]. Iron ores and metallurgical slags remain one of the necessary components of the burning of cement clinker [39]; thus, it should be assumed that the source of spherical particles is the cement and lime industry (marked with red colour). 

**Figure 4:**
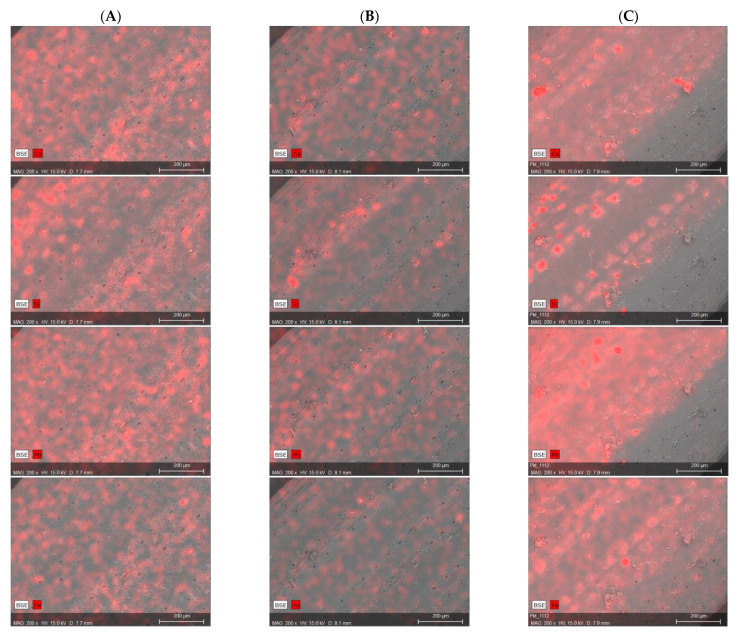
SEM microscopic images and energy dispersion spectroscopy (EDS) maps of Ca, Si, Pb, Fe distribution on the surface of needles; (**A**) Małogoszcz, (**B**) Bukowa, (**C**) Kowala.

The X-ray energy dispersion spectroscopy (EDS) confirmed the presence of lead, iron, aluminium, calcium, potassium, as well as magnesium in the surface layer of needles (Figure 4). The “glowing” areas of silica and calcium visible on the images are related to the stomata. 

The areas of concentration of lead and iron showed a considerable distance from the stomata and may prove their irregular distribution in the cellular structures of needles.

The elemental composition of the surface layer in the area shown on the image of the sample collected from Małogoszcz was arranged in a decreasing sequence Pb > Si > Ca > Fe > Al > K > Mg. The elements presented in the list are associated with the presence of anthropogenic pollutions. Because they did not regularly cover the entire analysed surface of the needle, their share has been shown as relatively small (the main elements detected in the EDS analysis were carbon and oxygen, deriving from vegetation material). 

In the sample from Bukowa, in turn, Si replaced its position with Ca in a decreasing order, whose share decreased from 1.9% to 0.9% (Si). A lower value in the sample from Bukowa in comparison to the sample from Małogoszcz was also found in the case of lead (from 2.5% to 1.5%), calcium (from 1.5% to 0.9%), iron and aluminium (from 0.7% up to 0.4%), and magnesium. 

In the sample from Kowala, a mutual change of position in a descending sequence representing the percentage of components was observed in the case of aluminium and iron (in relation to the sample from Małogoszcz).

These results are due to the location, topography, and meteorological conditions. The dominant wind directions in the study area are SW and W, which cause dispersion of pollutants on the surfaces of Małogoszcz and Kowala, and contribute to an increase in the recorded concentrations. In addition, an important element is the location of the surface in relation to hills and quarries. The presence of heavy metals (Fe, Al, Pb) on the surface of needles, determined by SEM/EDS, correlates with the content of heavy metals determined in the samples mineralised by means of ICP-MS.

## 4. Discussion

Tests of pH and chemical composition of pine needles carried out in 2018 in the Białe Zagłębie [30] confirmed their usefulness as a bioindicator of air pollution in the area of cement and limestone pressure. The results of the pH and chemical composition of the needles indicated intense alkaline pressure. For comparison, the research on *Pinus sylvestris* pine’s needles in the Słowiński National Park, which is considered the least polluted area in Poland [40], showed significantly lower concentrations of iron (109.7 mg·kg^−1^ d.m.) and copper (5.9 mg·kg^−1^ d.m.), comparable concentrations of manganese (211.1 mg·kg^−1^ d.m.), and higher concentrations of zinc (64.9 mg·kg^−1^ d.m.).

Research conducted in various areas of the world, using the SEM/EDS analyses, has proven the effectiveness of these methods in identifying pollutants in the assimilation organs of trees growing in the regions dominated by rock processing industry [41,42]. The EDS analysis of the needle surface allowed for determining the elemental composition (qualitative analysis) and the percentage of micro components (quantitative analysis). 

When interpreting the results of EDS analysis, the specificity of this analytical method should be taken into account. As a result of this analysis, graphic images are created in a parallel way with the SEM image of the sample’s surface. Information on the composition of the surface and the distribution of individual components are the result of measurements of the intensity and energy of X-rays, which is the effect of excitation of electrons of the electron cores of elements located on or near the surface of the material being tested. It should be remembered that the electrons used to create the SEM image (and when obtaining EDS data) penetrate the material to different depths depending on the type of matrix, but not more than for a few micrometers. This method is particularly suitable for imaging non-homogeneity in the distribution of elements on the surface of a given material; thus, it is a good tool for assessing the impact of dust on the surface of biological material. It also allows for indicating whether and how elements might migrate from pollutions and are absorbed by organic material. This analysis does not provide information on the elemental composition of deeper layers of material.

Additionally, based on the raw data concerning the X spectrum assigned to each pixel of the SEM image, quantitative data may be obtained, which allows for comparison of mass (or molar) shares of elements in the surface layer of the scanned area. For this purpose, a given area is selected for this analysis, rectangular or limited by a circle, inside which the obtained data on the X-ray intensity for individual wavelengths are integrated. Then, the data which have been integrated, are converted into a number of elements (all detected or selected, indicated by the operator), and results are further normalised so that the sum of shares amounts to 100%. When interpreting the quantitative results obtained in this way, the nature of the tested material, morphology and sources of origin of individual components of the sample, as well as the nature of the EDS analysis itself should be taken into account. Usually, data concerning the surface may not be directly compared to quantitative data obtained by other analytical techniques in which the entire mass of the sample is mineralised.

For the data presented below, the integration area was the entire image shown in the photographs in Figure 4. 

The analysis did not show any presence of lead in the needles from Kowala. Research on the chemical composition of dust from the cement plants in Małogoszcz and Nowiny was similar to the results obtained by Kozłowski in 2013 [10], although slight differences were conditioned by the variability of chemical composition of the input material. Nevertheless, the content of lead in the dust from the limestone plant in Trzuskawica (59 mg.kg^−1^) compared to that from Małogoszcz (164 mg.kg^−1^) constituted a clear difference, which was reflected in the elemental composition and the SEM/EDS image of needles from Małogoszcz and Kowala (remaining under the influence of the limestone plant in Trzuskawica). Prasad’s studies [43] have proven that the presence of heavy metals in needles may cause a reduction in a number of rows of stomata. According to the studies carried out by Strzyszcz [44], anthropogenic blast furnace particles with a diameter of 10 μm can travel up to 11 km, 5 μm particles—up to 44 km, and 2 μm—as much as 275 km. Considering the particle size depicted on the images, it may be stated that the direct source of heavy metal emissions in pine needles are primarily cement and limestone industry plants operating in this area. Nevertheless, the finer particles (including concentric spheres) are able to freely reach the area of Białe Zagłębie from considerable distances, which confirms the presence of iron-rich spheres on the surface of pine needles on the EDS images from the Silesia-Kraków industrial area [10,45]. 

## 5. Conclusions

SEM images of the needles of *Scots pine Pinus sylvestris* L. analysed in the area of operation of the cement and limestone plants of the Białe Zagłębie confirm the presence of particles characteristic of anthropogenic pollution. These particles may limit the possibility of gas exchange of pine assimilation organs by partial or complete sealing of stomata. 

EDS analysis confirmed the presence of Pb, Si, Ca, Al, K, and Mg on the surface and inside the cell structures of assimilation organs. It has been shown that the EDS analysis allows for associating the presence of individual elements in the analysed needle samples with their anthropogenic source of origins.

The images did not show a significant reduction in a number of rows of stomata, but a difference in the distance between them was found (two times the distance between the samples from Kowala and Bukowa).

## Figures and Tables

**Table 1:** Chemical composition of cement dust from Nowiny and Małogoszcz.

Cement Plant	Pb	Cd	Cr	Co	Cu	Mn	Ni	Zn	Sr	Mg	Al	Fe
	[mg·kg^−1^ d.m.]											
**Małogoszcz**	164.51	2.56	28.00	3.35	7.15	151.64	43.40	464.04	374.99	6786.85	29,004.18	17,480.63
**Nowiny**	134.70	1.85	22.04	6.75	21.29	428.19	51.37	849.23	287.62	12,269.10	45,217.87	23,029.20

## Data Availability

Not applicable.
